# Gain studies of 1.3-μm dilute nitride HELLISH-VCSOA for optical communications

**DOI:** 10.1186/1556-276X-7-526

**Published:** 2012-09-25

**Authors:** Faten Adel Ismael Chaqmaqchee, Naci Balkan

**Affiliations:** 1 School of Computer Science and Electronic Engineering, University of Essex, Colchester CO4 3SQ, UK; 2 Department of Physics, Faculty of Science and Health, University of Koya, Koya, KO50 1001, Iraq

**Keywords:** HELLISH, VCSOA, GaInNAs, Luminescence, Gain

## Abstract

The hot electron light emitting and lasing in semiconductor heterostructure-vertical-cavity semiconductor optical amplifier (HELLISH-VCSOA) device is based on Ga_0.35_In_0.65_ N_0.02_As_0.08_/GaAs material for operation in the 1.3-μm window of the optical communications. The device has undoped distributed Bragg reflectors (DBRs). Therefore, problems such as those associated with refractive index contrast and current injection, which are common with doped DBRs in conventional VCSOAs, are avoided. The gain versus applied electric field curves are measured at different wavelengths using a tunable laser as the source signal. The highest gain is obtained for the 1.3-μm wavelength when an electric field in excess of 2 kV/cm is applied along the layers of the device.

## Background

Currently, GaInNAs [1] quantum wells (QWs) grown on GaAs substrates are subject to wide interest, mainly for applications as vertical-cavity surface-emitting lasers and vertical-cavity semiconductor optical amplifiers (VCSOAs) for operation at the 1.3-μm wavelength region. These devices have numerous advantages over edge-emitting lasers and SOAs including less temperature sensitivity [2], high coupling efficiency to optical fiber (low noise figure), and low power consumption and cost. Furthermore, these structures allow for on-chip testing and fabrication of two-dimensional arrays. The VCSOAs have numerous applications including optical interconnects [3], wavelength-selective preamplifiers [4], and optical switches and modulators [5].

**Figure 1:**
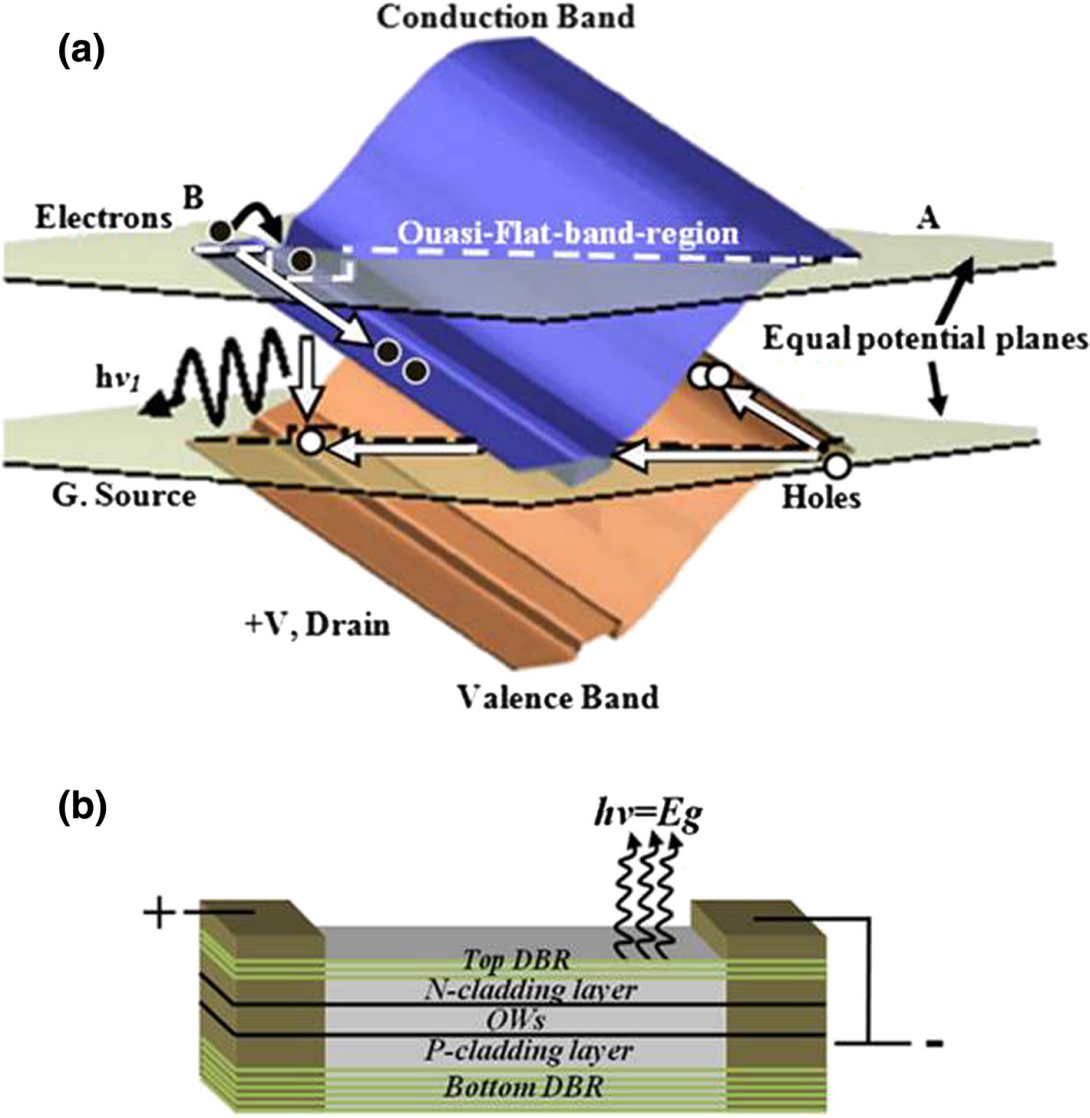
**The three-dimensional model for a standard HELLISH device.** (**a**) Schematic diagram showing the emission of light under quasi-flatband-region condition (adapted from [8]); (**b**) biasing of the two-terminal simple-bar device.

The hot electron light emitting and lasing in semiconductor heterostructure-vertical-cavity semiconductor optical amplifier (HELLISH-VCSOA) device is a surface emitter based on longitudinal injection of electron and hole pairs in their respective channels [6,7]. When the sample is illuminated in the absence of an external electric field, photogenerated carriers recombine radiatively in the QW without drifting along the longitudinal channels. When the device is biased, energy bands tilt upwards, where the degree of tilting is proportional to the applied voltage. At low bias, a quasi-flat region is established and a small number of carriers are able to drift diagonally into the p-n junction as illustrated in Figure 1. With an increase in the electric field, the energy bands will tilt up further, so more carriers flow into the active region, increasing the emitted light intensity.

**Figure 2:**
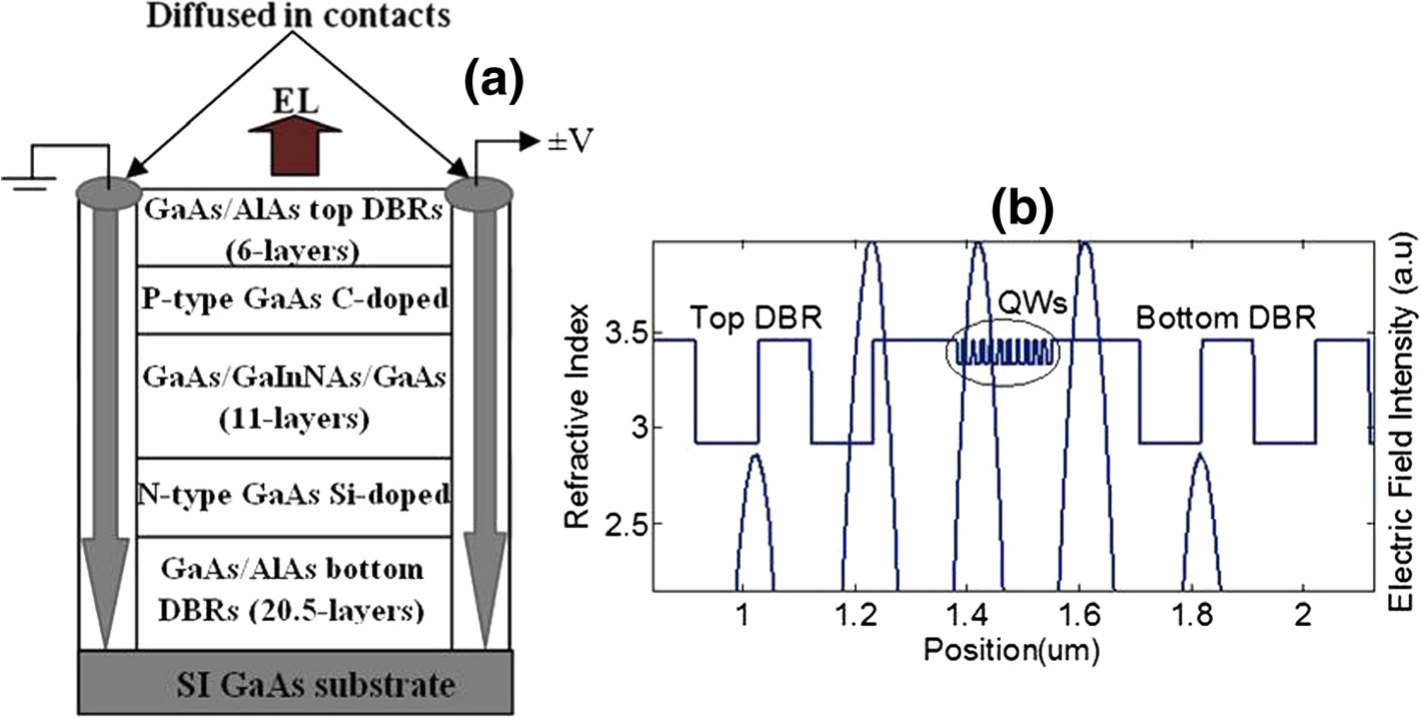
**Structure of HELLISH-VCSOA and refractive index profile and electric field intensity distribution.** (**a**) Schematic diagram to illustrate the structure of simple-bar HELLISH-VCSOA for light emission; (**b**) the refractive index profile and distribution of the electric field intensity across the sample.

The HELLISH-VCSOA device used in the experiments was grown by molecular beam epitaxy on a semi-insulating GaAs substrate. The structure of the device is shown in Figure 2a. The active layer consists of eleven 6-nm GaInNAs/GaAs QWs which are enclosed between two 150-nm-thick GaAs cladding layers. The doping layers are C-doped (top) and Si-doped (bottom). The MQWs are placed at the antinodes of the electric field in the 3*λ*/2 cavity to ensure maximum optical gain at the emission wavelength of 1.3 μm (Figure 2b). The distributed Bragg reflector (DBR) consists of 6 and 20.5 AlAs/GaAs quarter-wavelength pairs for the top and bottom mirrors, respectively. The estimated reflectivity of the top DBR is around 60%, while that of the bottom DBR is in excess of 99%. The device is fabricated in the form of a simple bar with a contact separation of 400 μm. A more detailed description of the structure, fabrication, and device operation can be found elsewhere [8].

The object of this work presented here is to achieve high gain at low applied voltages, thus low input powers. This is achieved by keeping the length of the devices short (typically 400 μm). The device is characterized at room temperature through the measurements of integrated electroluminescence (EL), light intensity-voltage characteristics, spectral photoluminescence (PL), EL, and electro-photoluminescence (EPL) techniques.

## Methods

**Figure 3:**
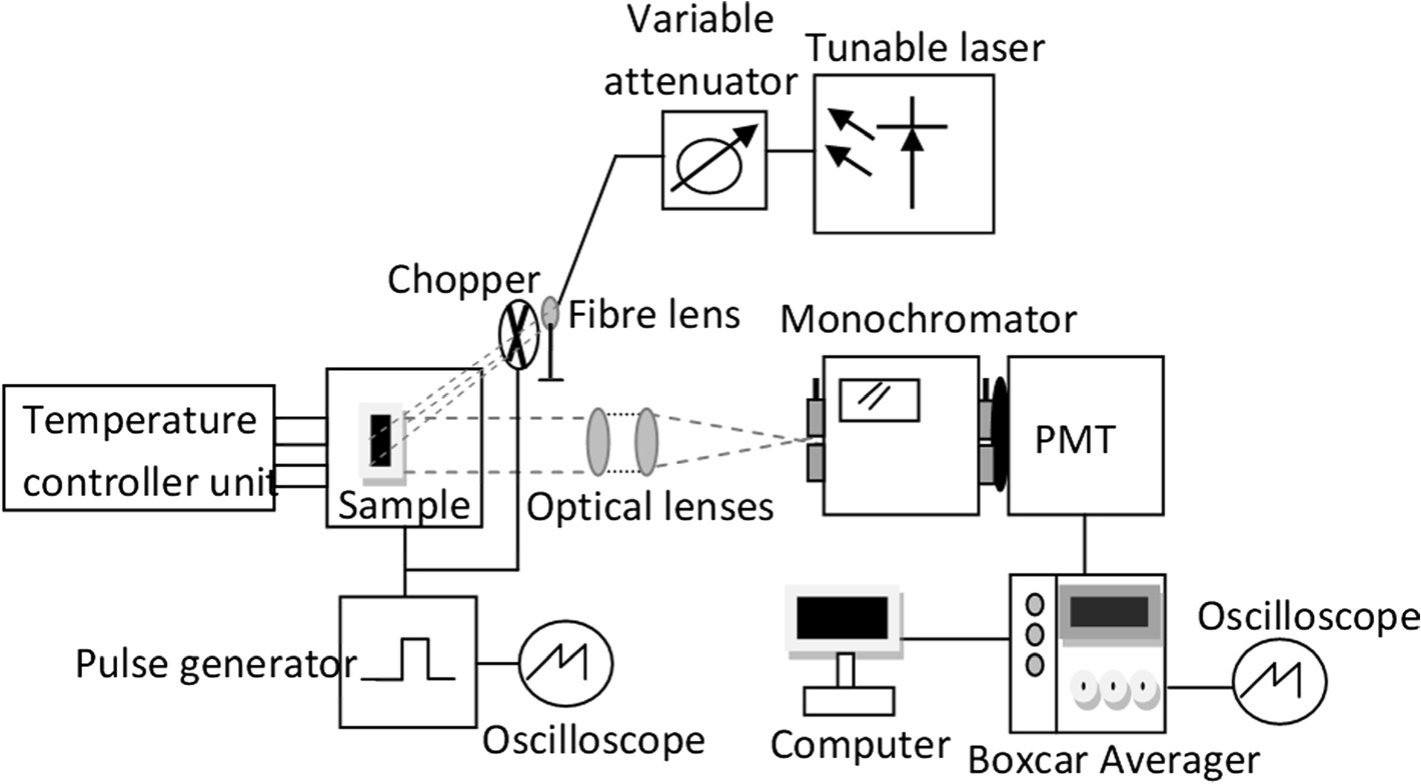
**Schematic diagram to illustrate the experimental setup for the device characterization at *T*** **= 20°C.**

The experimental setup to study the gain characteristics is shown in Figure 3. It consists of a NI PX1-1033 tunable laser (National Instruments Corporation, Austin, TX, USA) as the signal to be amplified where the wavelength and the output power can be tuned between 1,265 and 1,345 nm and between 1 and 6 mW, respectively. Optical collimator lens is used to send the laser signal through a mechanical optical chopper onto the sample. The sample is kept at 20°C using the TED-350 cooler (Thorlabs, Newton, NJ, USA). An AVTECH AVR-4B pulse generator (Avtech Electrosystems Ltd., Nepean, Canada) is used to apply the voltage pulses which are diffused in contacts along the layers. In order to avoid excessive Joule heating, the pulse width was kept below 20 μs and the duty cycle was less than 0.5%. The emission from the device is collected and dispersed by two optical lenses and a 1/3-m monochromator assembly. A cooled InGaAs photomultiplier is used to convert incoming light signals into electrical signals. The latter is then enhanced using the Model 165 Gated Integrator and Model 162 Boxcar Averager module (EG&G Princeton Applied Research, Princeton, NJ, USA) and a computer system.

## Results and discussions

**Figure 4:**
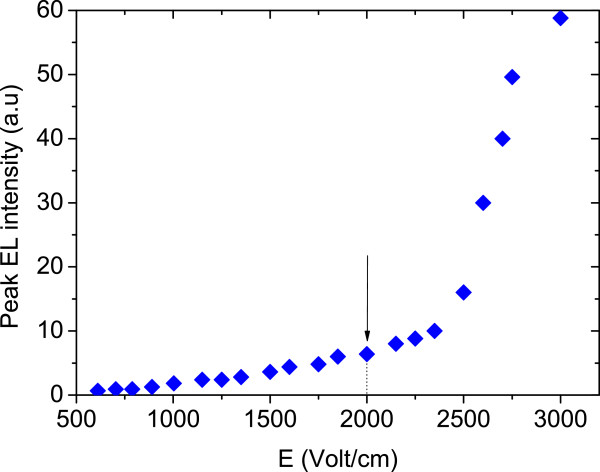
**Plot of the integrated EL intensity versus the applied electric fields (*E*) at room temperature.** The arrow indicates that the sample starts to illuminate at around *E* = 2 kV/cm.

**Figure 5:**
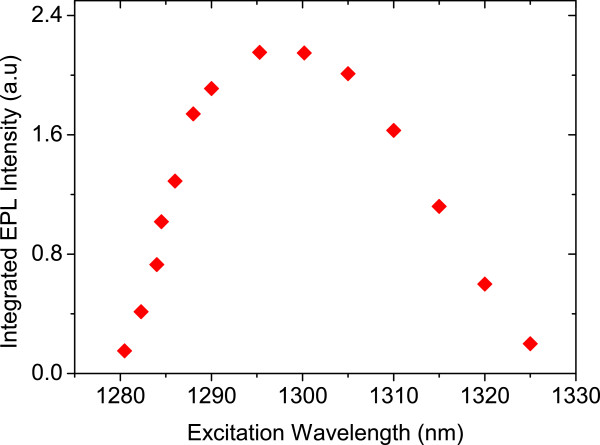
**Integrated EPL intensity against excitation wavelength.** This was measured at an applied electric Fields of *E* = 2 kV/cm and an output laser power of *P* = 6 mW.

The plot of the integrated EL intensity versus the applied electric field is shown in Figure 4. It is clear from Figure 4 that there is a transition from the spontaneous emission to lasing at an electric field of approximately *E* = 2 kV/cm. The EL tends to saturate at *E* > 3 kV/cm. The saturation is probably due to the Joule heating. Figure 5 shows the integrated EPL intensity when the device is biased with 80 V (*E* = 2 kV/cm). Here, the tunable laser power is 6 mW, and its wavelength is varied between *λ* = 1,280 and 1,325 nm. The integrated EPL intensity has a broad peak at around *λ* = 1,300 nm that corresponds to the maximum gain wavelength.

**Figure 6:**
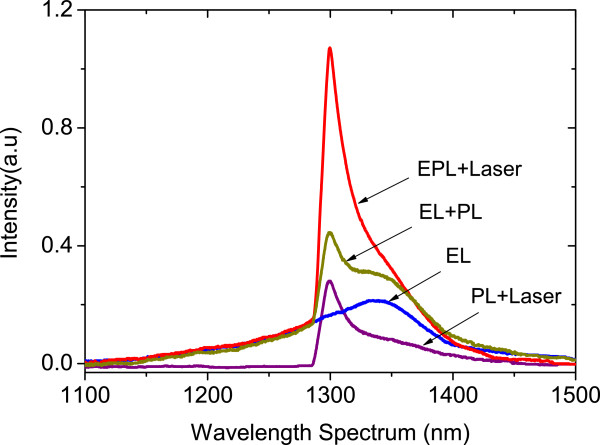
**Measured PL + laser, EL, sum (EL + PL and laser), and EPL + laser spectra.** This was through an applied electric field of *E* = 2 kV/cm and an excitation wavelength of *λ* = 1.3 μm at *T* = 20°C.

**Figure 7:**
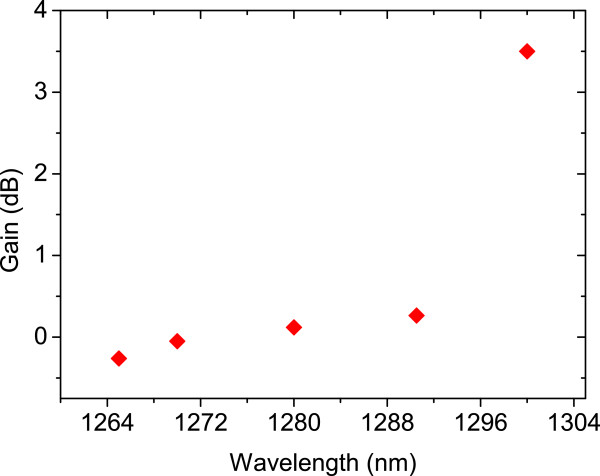
**The peak gain versus excitation wavelengths at *T*** **= 20°C.**

The PL, EL, and the combined EPL spectra are shown in Figure 6. In PL, the optical CW signal of the 1,300-nm wavelength is sent onto the device under excitation by low power of less than 1 mW. In EL, the device is externally biased with an electric field of 2 kV/cm. The EPL spectrum is measured by combining the two techniques of PL and EL. As shown in Figure 7, the gain from the device was determined by subtracting the sum (EL + PL) peak intensity from the EPL and divided by the PL. The peak gain was significantly increased with tunable laser wavelength. It has reached a maximum point of around 4 dB at *λ* = 1,300 nm.

## Conclusions

We report finite gain from an electrically pumped HELLISH-VCSOA at a wavelength of *λ* = 1,300 nm. The gain may be improved by a better control of Joule heating at high electric fields.

## Abbreviations

*λ*: wavelength; DBRs: distributed Bragg reflectors; *E*: electric fields; EL: electroluminescence; EPL: electro-photoluminescence; HELLISH: hot electron light-emitting and lasing in semiconductor heterostructure; PL: photoluminescence; QWs: quantum wells; *T*: temperature; VCSOA: vertical-cavity semiconductor optical amplifier.

## Competing interests

The authors declare that they have no competing interests.

## Authors’ contributions

NB and FAI designed the structure. FAI fabricated the device, carried out the experiments work, and wrote up the article. NB is the inventor of the original device and the overall supervisor of the project. Both authors read and approved the final manuscript.
